# The Inhibitory Core of the Myostatin Prodomain: Its Interaction with Both Type I and II Membrane Receptors, and Potential to Treat Muscle Atrophy

**DOI:** 10.1371/journal.pone.0133713

**Published:** 2015-07-30

**Authors:** Yutaka Ohsawa, Kentaro Takayama, Shin-ichiro Nishimatsu, Tadashi Okada, Masahiro Fujino, Yuta Fukai, Tatsufumi Murakami, Hiroki Hagiwara, Fumiko Itoh, Kunihiro Tsuchida, Yoshio Hayashi, Yoshihide Sunada

**Affiliations:** 1 Department of Neurology, Kawasaki Medical School, Kurashiki, Okayama, 701–0192, Japan; 2 Department of Medicinal Chemistry, Tokyo University of Pharmacy and Life Sciences, Hachioji, Tokyo, 192–0392, Japan; 3 Department of Molecular and Developmental Biology, Kawasaki Medical School, Kurashiki, Okayama, 701–0192, Japan; 4 National Research Center for Protozoan Diseases (NRCPD), Obihiro University of Agriculture and Veterinary Medicine, Obihiro, Hokkaido, 080–8555, Japan; 5 Department of Health and Sports Sciences, Faculty of Medical Professions, Kawasaki University of Medical Welfare, Kurashiki, Okayama, 701–0193, Japan; 6 Department of Medical Science, Teikyo University of Science, Uenohara, Yamanashi, 409–0193, Japan; 7 Laboratory of Cardiovascular Medicine, Tokyo University of Pharmacy and Life Sciences, Hachioji, Tokyo, 192–0392, Japan; 8 Division for Therapies against Intractable Diseases, Institute for Comprehensive Medical Science, Fujita Health University, Toyoake, Aichi, 470–1192, Japan; Institut de Myologie, FRANCE

## Abstract

Myostatin, a muscle-specific transforming growth factor-β (TGF-β), negatively regulates skeletal muscle mass. The N-terminal prodomain of myostatin noncovalently binds to and suppresses the C-terminal mature domain (ligand) as an inactive circulating complex. However, which region of the myostatin prodomain is required to inhibit the biological activity of myostatin has remained unknown. We identified a 29-amino acid region that inhibited myostatin-induced transcriptional activity by 79% compared with the full-length prodomain. This inhibitory core resides near the N-terminus of the prodomain and includes an α-helix that is evolutionarily conserved among other TGF-β family members, but suppresses activation of myostatin and growth and differentiation factor 11 (GDF11) that share identical membrane receptors. Interestingly, the inhibitory core co-localized and co-immunoprecipitated with not only the ligand, but also its type I and type II membrane receptors. Deletion of the inhibitory core in the full-length prodomain removed all capacity for suppression of myostatin. A synthetic peptide corresponding to the inhibitory core (p29) ameliorates impaired myoblast differentiation induced by myostatin and GDF11, but not activin or TGF-β1. Moreover, intramuscular injection of p29 alleviated muscle atrophy and decreased the absolute force in caveolin 3-deficient limb-girdle muscular dystrophy 1C model mice. The injection suppressed activation of myostatin signaling and restored the decreased numbers of muscle precursor cells caused by caveolin 3 deficiency. Our findings indicate a novel concept for this newly identified inhibitory core of the prodomain of myostatin: that it not only suppresses the ligand, but also prevents two distinct membrane receptors from binding to the ligand. This study provides a strong rationale for the use of p29 in the amelioration of skeletal muscle atrophy in various clinical settings.

## Introduction

Myostatin, a muscle-specific transforming growth factor-β (TGF-β) family member, plays crucial roles in negative regulation of skeletal muscle mass [1]. Similar to certain other TGF-β family members, myostatin is synthesized as a precursor, dimeric protein consisting of an N-terminal prodomain and C-terminal mature domain (ligand) [2,3]. After processing by furin-like proteases, the N-terminal prodomain noncovalently binds to the C-terminal ligand and forms an inactive latent complex to suppress its biological activities in circulation [3]. Upon cleavage of the prodomain by bone morphogenetic protein (BMP)-1/tolloid family of metalloproteinases, the ligand recruits and phosphorylates two distinct membrane serine/threonine receptors, termed type I and II, which in turn activate the intracellular effector molecule Mad homolog (Smad) 2 and Smad3, and subsequent Smad-responsive gene transcription [4,5]. Thus, the prodomain appears to be a crucial physiological inhibitor of the biological activity of myostatin [3]. The prodomain possesses the cleavage site for BMP-1/tolloid family of metalloproteinases [4], and the putative binding site for thrombospondin-1 (TSP-1), a major activator of the TGF-β1 ligand in recruitment of membrane receptors [6]. However, the regions critical for suppression of the myostatin signal have remained unknown.

Caveolin 3, a muscle-specific integral membrane protein, forms caveolae and functions as a scaffold protein by binding and regulating several signaling molecules such as Src tyrosine kinases, epidermal growth factor receptor, and G-proteins [7]. Heterozygous mutations in the *CAV3* gene give rise to limb-girdle muscular dystrophy (LGMD) 1C characterized by severe deficiency of caveolin 3 protein in muscle fibers [8]. We generated transgenic mice expressing Pro104Leu mutant caveolin 3 (CAV3^P104L^). These LGMD1C model mice developed muscle atrophy with loss of caveolin 3, indicating a dominant-negative effect of the mutant caveolin 3 [9]. We found that activated type I receptor and subsequent intramuscular myostatin signaling in the caveolin 3-deficient atrophic muscles was ameliorated by genetic introduction of the full-length myostatin prodomain [10].

In the current study, we identified the inhibitory core in the prodomain required to suppress myostatin signaling by expressing various prodomain regions as Fc fusion proteins in assayed cells. We also explored the ability of the corresponding peptide to enhance myogenesis *in vitro* by addition to the culture medium of differentiating myoblasts and to increase muscle mass or ameliorate muscle atrophy *in vivo* by intramuscular injection into caveolin 3-deficient LGMD1C mice or their wild-type littermates. This study provides the basis for future peptide therapy of patients with muscular atrophy.

## Materials and Methods

### Plasmid vectors

To express various prodomain regions as Fc fusion proteins, the cDNA of each prodomain region was amplified by RT-PCR from human skeletal muscle mRNA and subcloned into the pcDNA3-hFc vector that harbors the human IgG1 Fc region at the C-terminus [11]. For immunoprecipitation, RT-PCR products of the C-terminal FLAG-tagged inhibitory core of the prodomain and C-terminal V5- or HA-tagged ligand or receptor were subcloned into the pCS2+ expression vector.

### Luciferase assay

The pGL3-(CAGA)_12_-luciferase reporter gene contains the Smad-binding sequence (CAGA) [12]. HEK293 human embryonic kidney cells (Riken-BRC, Wako, Saitama, Japan) and A204 human rhabdomyosarcoma cells (ATCC, Manassas, VA, USA) were grown in Dulbecco’s modified Eagle’s medium (DMEM) and McCoy 5a medium supplemented with 10% fetal bovine serum (FBS), respectively. Cells were seeded in 12-well plates at 24 h before transfection with the pGL3-(CAGA)_12_-luciferase reporter gene, pCMV-β-gal, and either the empty vector (pcDNA3-hFc) or pcDNA3-hFc harboring various prodomain regions as human Fc fusion proteins. After 24 h, the medium was replaced with serum-free DMEM containing 10 ng/ml recombinant myostatin, 10 ng/ml recombinant growth and differentiation factor 11 (GDF11), 6 ng/ml recombinant TGF-β1, or 10 ng/ml recombinant activin A (R&D Systems, Minneapolis, MN, USA). After an additional 24 h of culture, the cells were processed and their luciferase activity was measured by a luciferase reporter assay system (Promega, Madison, WI, USA). All experiments were performed in triplicate, repeatedly at least twice. Values were normalized to β-galactosidase activity as described previously [10,13].

The expression of fusion proteins was confirmed by immunoblot analysis using a goat anti-human Fc antibody (Jackson ImmunoResearch Labs, West Grove, PA, USA).

### Co-localization and co-immunoprecipitation assay

COS-7 monkey kidney cells (Riken-BRC) were co-transfected with expression vectors bearing the FLAG-tagged inhibitory core of the prodomain with the V5- or HA-tagged ligand or receptor. All transfections were carried out in triplicate and the experiment was performed at least twice. The cells were fixed, permeabilized, and stained with an anti-rabbit FLAG polyclonal antibody (F7425; Sigma-Aldrich, St. Louis, MO, USA) and anti-mouse V5 monoclonal antibody (mAb) (R960-25; Invitrogen, Carlsbad, CA, USA) or anti-mouse HA mAb (12CA5; Roche Diagnostics, Indianapolis, IN, USA) for 60 min. Then, the cells were incubated with a fluorescent anti-rabbit and anti-mouse IgG secondary antibody (Invitrogen). The stained cells were visualized and imaged using a confocal laser microscope (TCS SP2, Leica Microsystems, Buffalo Grove, IL, USA).

Whole cell extracts were prepared by incubation in lysis buffer (50 mM Tris-HCl, pH 7.4, 50 mM NaCl, 1% Triton X-100, and 100 mM octyl glucoside) with a proteinase inhibitor cocktail on ice for 30 min as described previously [10]. After centrifugation at 10,000 × *g* for 30 min, the supernatant was incubated with an anti-FLAG mAb (M2, Sigma-Aldrich), anti-V5 mAb, or anti-HA mAb agarose gel to obtain immunoprecipitants as described previously [10].

### *In vitro* myogenic differentiation assay

C2C12 mouse myoblasts (Riken-BRC) expressing myostatin, GDF11, activin A, TGF-β1, or Pro104Leu mutant caveolin 3 were generated using a retroviral system as described previously [13,14]. Mononucleated C2C12 myoblasts grown in DMEM supplemented with 10% FBS were induced to differentiate into multinucleated myotubes in differentiation medium consisting of DMEM supplemented with 2% horse serum (HS). The cells were subjected to Wright-Giemsa post-staining to evaluate fusion indices as described previously [13]. For immunocytochemical analyses, the cells were fixed, permeabilized, and stained with antibodies against anti-myosin heavy chain (MY-32; Sigma-Aldrich), muscle creatine kinase-M (N-13, Santa Cruz Biotechnology, Santa Cruz, CA, USA), or myogenin (F5D, Santa Cruz Biotechnology), followed by an Alexa 488-conjugated secondary antibody (Invitrogen). Cell lysates were size-fractionated by SDS-polyacrylamide gel electrophoresis and immunoblotted using MY-32. These infection experiments were carried out in triplicate, repeatedly twice.

### Experimental animals

All animal experiments were approved by the Recombinant DNA Experiments Safety Committee (#13–04) and Animal Research Committee (#13–081) of Kawasaki Medical School. CAV3^P104L^ and wild-type littermate mice were maintained (*n* = 5 per cage) at 22°C under a 12:12 h light/dark cycle with free access to water and standard laboratory food (CE-2, CLEA Japan Inc., Fuji, Shizuoka, Japan). The water and food intake of the mice was monitored daily, and their body weights were recorded weekly. Muscles were isolated following euthanasia under sevoflurane-induced anesthesia.

### Intramuscular injection of a synthetic peptide

A peptide corresponding to the 29-amino acid inhibitory core (p29) of mouse myostatin was synthesized and purified to >95% as assessed by high-performance liquid chromatography (KNC Laboratories, Kobe, Hyogo, Japan). The peptide (5–20 nmol) was injected into the ipsilateral tibialis anterior (TA) muscle of male caveolin 3-deficient transgenic mice (CAV3^P104L^) [8,9] or wild-type littermate mice aged 12 weeks (*n* = 10) under inhalational anesthesia with sevoflurane. The same amount of albumin was injected into the contralateral TA muscle as a control. The specific tetanic force of the isolated TA muscles from the mice was measured as described previously [13]. Peak grip strength (g) was measured with a grip strength meter (MK-380S, Muromachi, Bunkyou-ku, Tokyo, Japan). At 1 month after peptide injection, the TA muscles were isolated, and the tetanic force, single myofiber area (SMA), satellite cell number, phosphorylated (p)-Smad2 level, and *Cdkn1a* ^*p21*^ or *Cdkn2b* ^*p15*^ gene expression were measured as described previously [10,13]. Single myofibers were isolated from the TA muscles as described previously [13,15]. Isolated myofibers were fixed, permeabilized, and stained for a satellite cell marker, caveolin 1 (BD Transduction Laboratories, Lexington, KY, USA) [16]. Nuclei were counterstained with 4′, 6-diamidino-2 phenylindole (DAPI) provided in the mounting media (Vectashield, Vector laboratories, Burlingame, CA, USA). Myofiber type immunohistochemistry was performed using monoclonal antibodies (Developmental Studies Hybridoma Bank, Iowa, IA, USA) against type I (BA-D5), type IIA (SC-71), and type IIB (BF-F3) myosin heavy chains and appropriate Alexa 594-conjugated secondary antibodies (Invitrogen).

### Statistical analysis

Statistical analyses were performed using one-way analysis of variance followed by Bonferroni’s test. *P-*values of less than 0.05 were considered statistically significant.

## Results

### Identification of the inhibitory core of the myostatin prodomain

**Fig 1:**
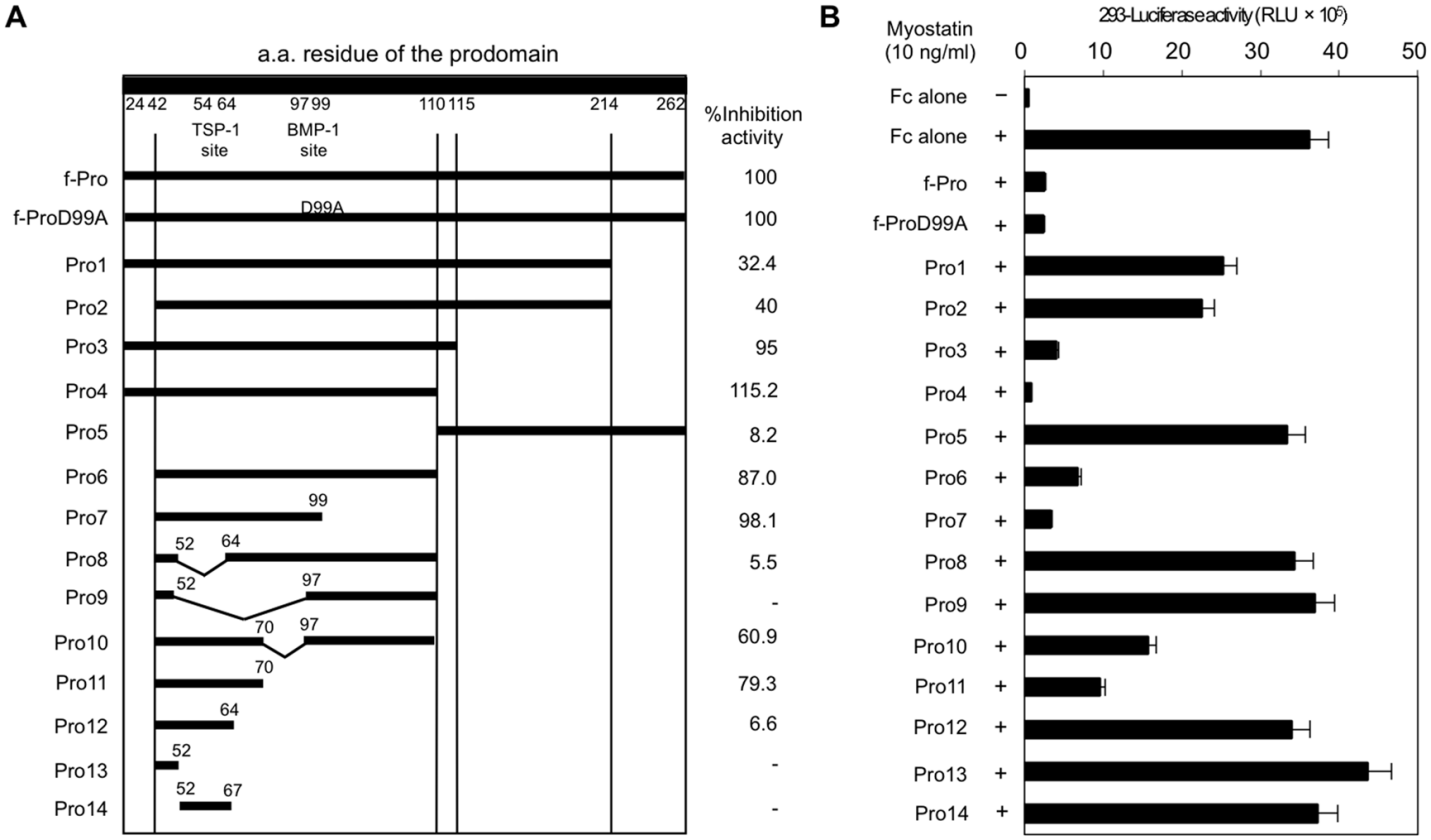
Identification of the inhibitory core of the myostatin prodomain. (**A**) Truncation and deletion constructs of human myostatin prodomain:human Fc fusion proteins (**left**). Percentage inhibitory effect of each construct on myostatin activity in comparison with the full-length prodomain (f-Pro, **right**). (**B**) Recombinant myostatin-induced transcriptional activity in HEK293 human embryonic kidney cells co-transfected with a pGL3-(CAGA)_12_-luciferase reporter gene, pCMV-β-Gal, and various prodomain region:Fc fusion constructs. Values are the mean ± SD (*n* = 6). RLU, relative luminescence units.

We designed a series of truncation and deletion constructs of the full-length human prodomain including 239 amino acid residues (^24^N–K^262^). We considered two known sites:, ^54^EAIKIQILSKL^64^, the putative binding site for TSP-1 [6], and ^97^QRD^99^, the cleavage site for BMP-1/tolloid family of metalloproteinases [4] (Fig 1A). Each construct was co-transfected with a TGF-β-sensitive Smad-responsive luciferase reporter gene into HEK293 human embryonic kidney cells. To evaluate *in vitro* myostatin activities, luciferase reporter assays were performed in triplicate, repeatedly at least twice. Stimulation of cells carrying an empty Fc vector with recombinant myostatin ligand significantly increased luciferase activity above the basal level (Fig 1B). Expression of the full-length prodomain:Fc fusion protein (f-Pro) resulted in a significant reduction of myostatin-induced transcriptional activity. Consistent with a previous report [4], a mutant (^97^QAD ^99^) full-length prodomain:Fc fusion protein (f-ProD99A) was as effective at blocking myostatin-induced transcriptional activity as f-Pro. Additionally, f-ProD99A showed no proteolytic degradation products in immunoblot analysis (S1 Fig). We thus performed the following assays without considering the effect of cleavage by BMP-1/tolloid family of metalloproteinases.

Co-transfection of each truncated prodomain:Fc fusion protein suppressed luciferase activity in a region-specific manner (Fig 1). Compared with f-Pro, the N-terminal half of the prodomain (Pro4) showed a significantly increased capacity for inhibition of myostatin-induced transcriptional activity. In contrast, the C-terminal half of the prodomain (Pro5) lacked the inhibitory effect on myostatin-induced transcription. We thus assumed that the inhibitory core of the prodomain was located in the N-terminal half. We constructed expression vectors focusing on the N-terminal half including Pro6-14. As shown in Fig 1, Pro11, lacking the 19 N-terminal and 40 C-terminal amino acids of Pro4, showed 79% inhibitory activity compared with f-Pro. The inhibitory activity by Pro11 was higher than those by Pro8, Pro9, Pro10; neighborhood deletion constructs in consideration with the TSP-1 site and the BMP-1 site. Further deletion constructs from Pro11, including Pro12-14 also showed lower inhibitory effects on myostatin activity compared with Pro11. The inhibitory effect of Pro11 was consistent in A204 human rhabdomyosarcoma cells (S2 Fig). We conclude that Pro11 consisting of 29 amino acid residues is the inhibitory core of the myostatin prodomain.

### The inhibitory core of the myostatin prodomain preferentially inhibits the biological activity of myostatin and its analog, GDF11

**Fig 2:**
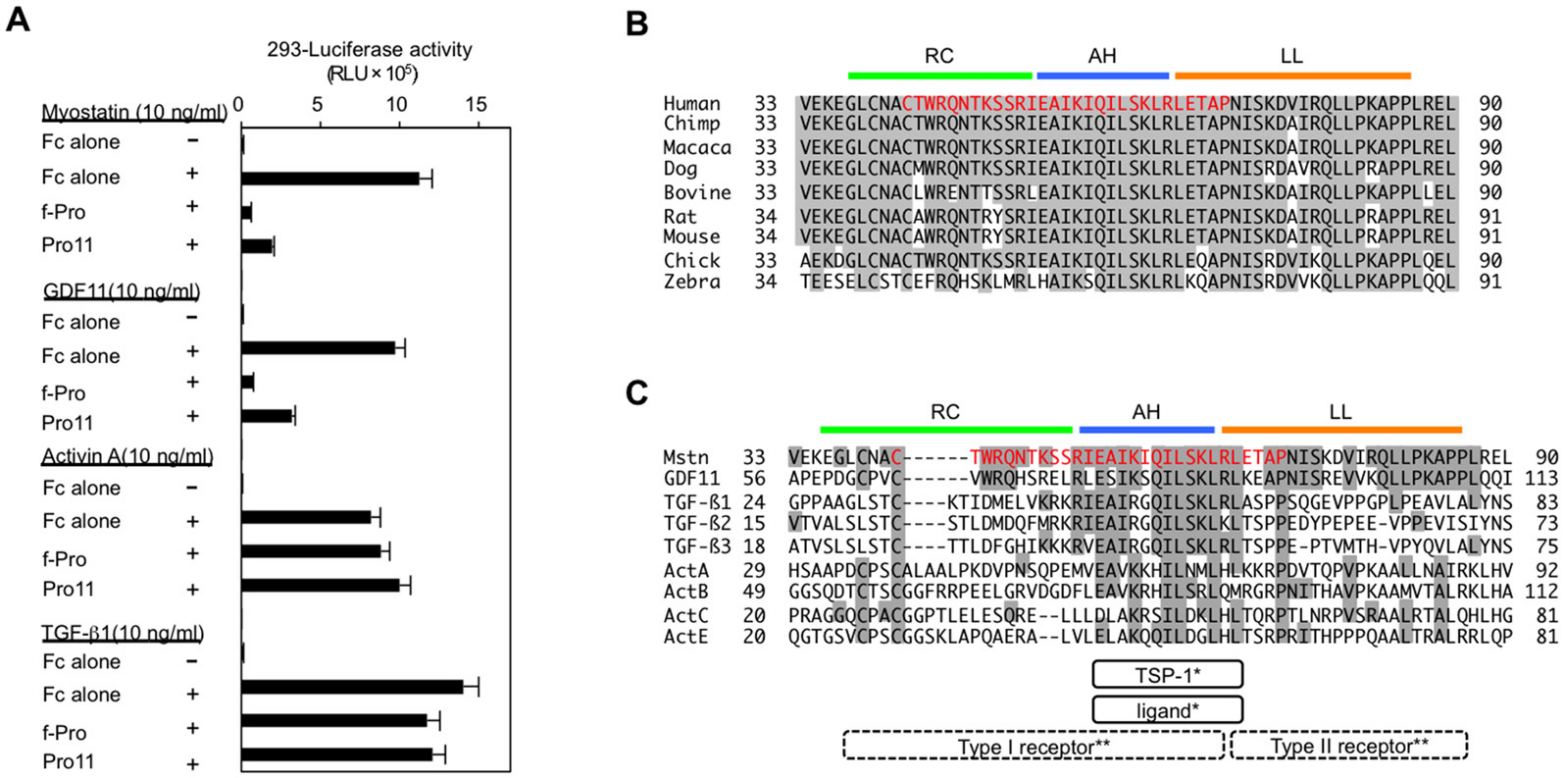
The identified inhibitory core of the myostatin prodomain specifically suppresses myostatin and its analog, GDF11, and includes an AH that is evolutionarily conserved among several other TGF-β family members. (**A**) The full-length myostatin prodomain (f-Pro) and its inhibitory core (Pro11) inhibited the transcriptional activities of myostatin and GDF11, but not of TGF-β1 or activin A, in HEK293 cells. (**B, C**) Sequence alignment of the prodomains of myostatin in nine species (**B**) and nine TGF-β family members (**C**). Red indicates the identified inhibitory core of the myostatin prodomain, consisting of 29 amino acids. The AH structure (blue) of the TGF-β1 prodomain has been shown to bind to both its ligand and TSP-1* [6,18]. Crystallographic analyses of TGF-β1 and its receptors have predicted that the random coiled structure (RC, green) and the AH are located closely to its type I receptor, whereas the latency lasso structure (LL, brown) is located close to its type II receptor** [19,20].

A previous study revealed that the full-length myostatin prodomain specifically suppresses myostatin and its analog, GDF11, but not activin [17]. We thus examined which TGF-β family members can be suppressed by the identified inhibitory core of the myostatin prodomain *in vitro*. As shown in Fig 2A, the inhibitory core (Pro11) inhibited not only myostatin, but also GDF11 to the same extent. In contrast, the activities of activin and TGF-β1 were not affected by the inhibitory core, suggesting certain specificity of the inhibitory core for suppression of TGF-β family members.

### The inhibitory core of the prodomain suppresses myostatin activity by interacting with myostatin ligand and two distinct receptors

By creating a multiple sequence alignment from a protein BLAST, we found that the 29-amino acid inhibitory core in the human myostatin prodomain was well conserved not only in other species (Fig 2B), but also among other TGF-β family members (Fig 2C). Of note, a recent mutational analysis demonstrated that the corresponding region in the TGF-β1 prodomain includes an α-helix (AH) that binds to and suppresses the TGF-β1 ligand [18]. As shown in Fig 2C, the AH almost corresponds to the TSP-1 site. Importantly, more recent three-dimensional crystallographic analyses predicted that the inhibitory core of TGF-β1 was closely located to both type I and II receptors for TGF-β1 and the TGF-β1 ligand [19,20].

**Fig 3:**
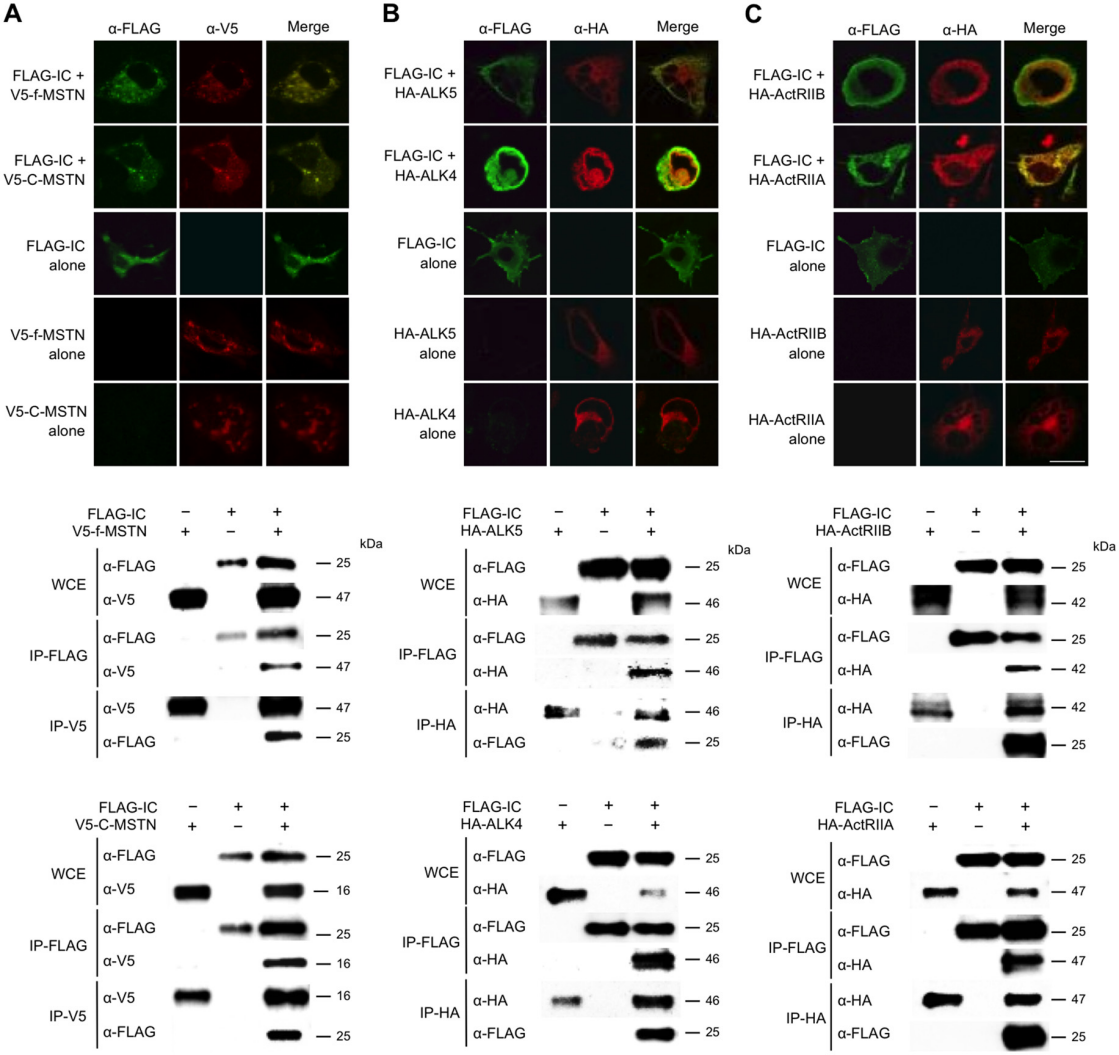
Interaction of the inhibitory core of myostatin with its ligand and receptors. Co-localization (**Upper**) and co-immunoprecipitation (**Lower**) of the inhibitory core (IC) of the myostatin prodomain and its ligand (**A**), its type I receptors (ALK4 and ALK5, **B**), and its type II receptors (ActRIIA and ActRIIB, **C**) in COS-7 embryonic kidney cells expressing FLAG-tagged IC and V5- or HA-tagged ligand or receptors. Scale bar, 20 μm. Whole cell extracts (WCE) were immunoprecipitated with anti-FLAG, anti-V5, or anti-HA agarose and then immunoblotted using anti-FLAG, anti-V5, or anti-HA antibodies, respectively.

To explore the precise molecular mechanism by which the inhibitory core of the myostatin prodomain suppresses the biological activity of myostatin, we first examined whether the inhibitory core of the prodomain can interact with the type I and II receptors, and the myostatin ligand. COS-7 monkey kidney cells were co-transfected with constructs harboring the C-terminal FLAG-tagged inhibitory core and V5-tagged full-myostatin (prodomain + ligand) or C-terminal mature myostatin (ligand). As expected, individually tagged proteins co-localized and co-immunoprecipitated with each other (Fig 3A). Similar results were observed for the FLAG-tagged inhibitory core and HA-tagged full-GDF11 (prodomain + ligand) or C-terminal mature GDF11 (ligand) (S3 Fig). To further explore the intriguing interactions of the inhibitory core with receptors, we co-transfected COS-7 cells with constructs harboring a FLAG-tagged inhibitory core and either HA-tagged type I (ALK5, ALK4) or type II (ActRIIB, ActRIIA) receptors [21]. Fig 3 shows co-localization and co-immunoprecipitation of the inhibitory core and each receptor (Fig 3B and 3C). These results suggest that the identified inhibitory core can interact not only with its ligand, but also the two receptors as previously predicted by TGF-β1 crystal structure analyses [19,20].

**Fig 4:**
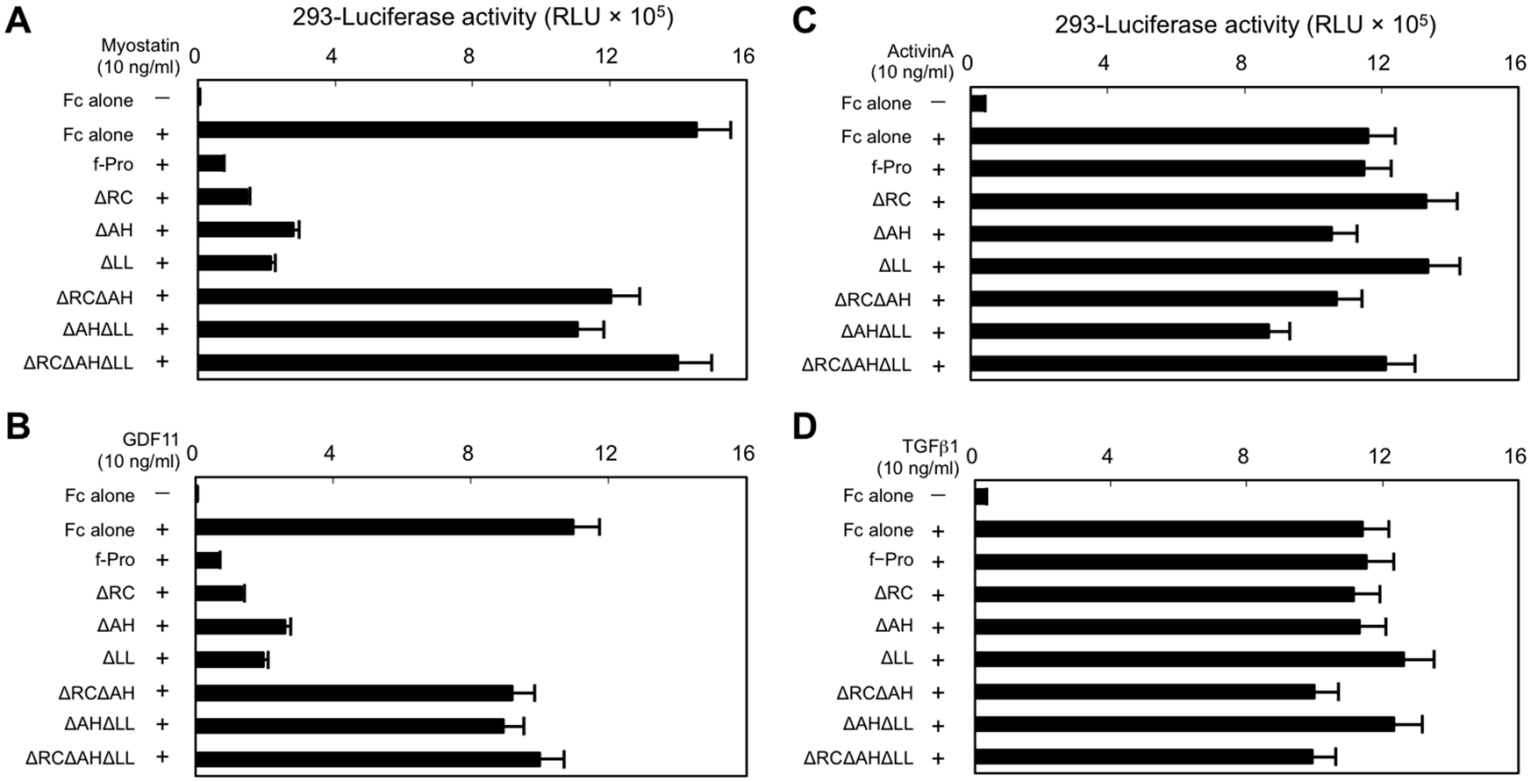
Three consecutive regions within the inhibitory core exhibit coordinated suppression of myostatin activity. Constructs (ΔRC, ΔAH, and ΔLL) with single deletions of the RC, AH, or LL regions of full-length myostatin (f-Pro) showed an inhibitory effect on the transcriptional activities of myostatin (**A**) and GDF11 (**B**), but not activin A (**C**) or TGF-β1 (**D**), in HEK293 cells. In contrast, combined deletion constructs (ΔRCΔAH, ΔAHΔLL, and ΔRCΔAHΔLL) had no inhibitory effect on myostatin (**A**) or GDF11 (**B**) activation in the HEK293-(CAGA)_12_-luciferase system.

Next, we divided the 29-amino acid inhibitory core of the myostatin prodomain into three sections according to previous descriptions [18,19,20]: the N-terminal random coiled structure (RC, ^42^CTWRQNTKSSRI^53^), AH structure (AH, ^54^EAIKIQILSKL^64^), and C-terminal latency lasso structure (LL, ^65^RLETAP^70^). The AH has been shown to correspond to the binding site of the TGF-β1 prodomain for its ligand [18]. We constructed deletion constructs of RC, AH, or LL in the full-length prodomain and compared their inhibitory effects on myostatin signaling in HEK293 cells. As shown in Fig 4A, single deletion constructs (ΔRC, ΔAH, and ΔLL) significantly inhibited myostatin activity compared with f-Pro. However, combined deletion constructs (ΔRCΔAH, ΔAHΔLL, and ΔRCΔAHΔLL) lost their inhibitory activities, indicating that the three regions in the inhibitory core coordinately suppress myostatin activity by interacting with the receptors and ligand. Similar results were observed for GDF11 (Fig 4B), but not activin (Fig 4C) or TGF-β1 (Fig 4D).

### A synthetic peptide corresponding to the inhibitory core, restores impaired myoblast differentiation induced by myostatin and GDF11, but not activin A or TGF-β1

**Fig 5:**
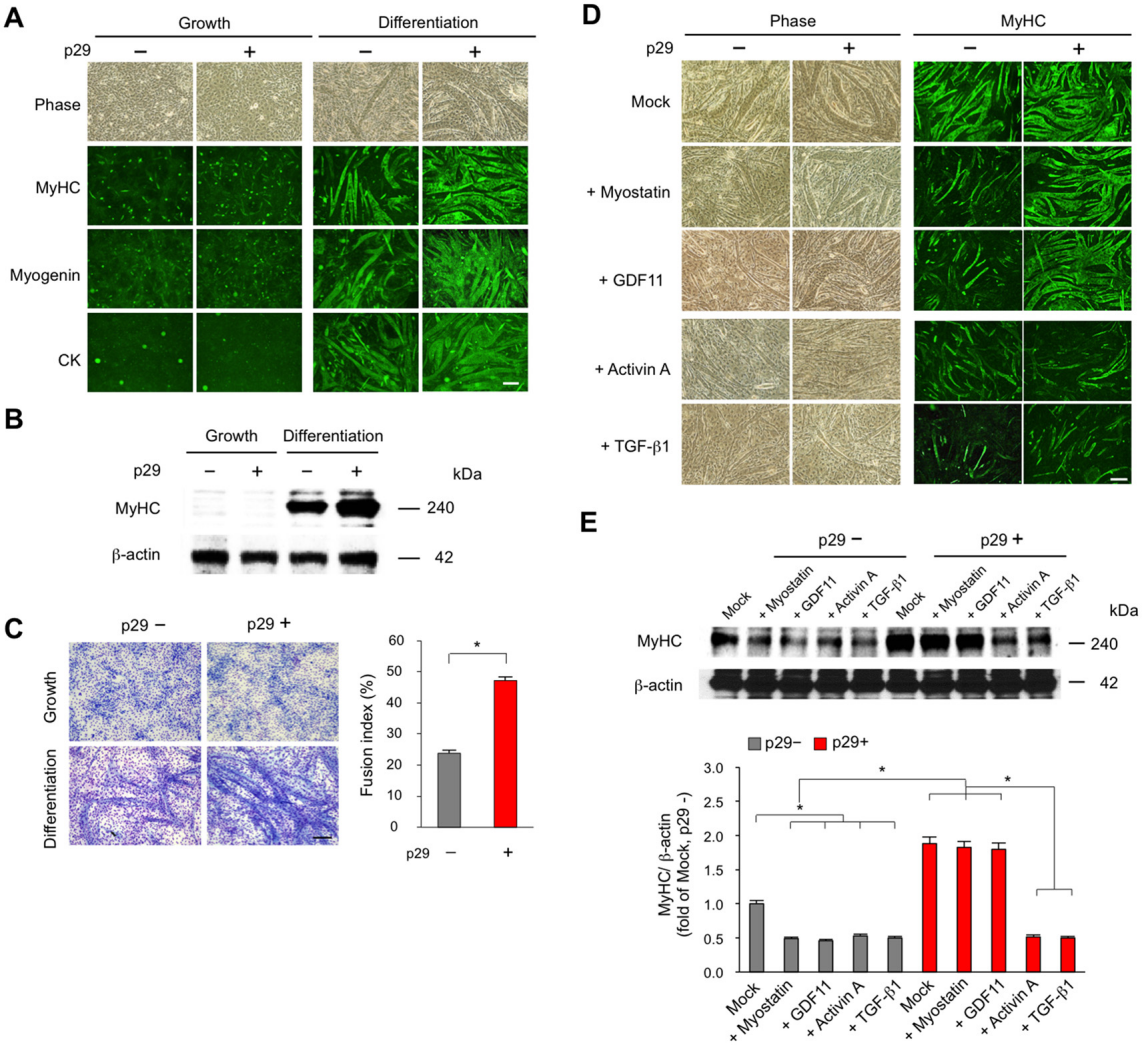
p29 enhances myogenesis suppressed by myostatin and GDF11, but not activin or TGF-β1. (**A**) C2C12 myoblasts were maintained in growth medium. Mononucleated myoblasts differentiate into multinucleated myotubes in differentiation medium with (+) or without (–) 1 μM p29 for 7 days. Phase-contrast and fluorescence images of cells stained for myotube markers MyHC, myogenin, and CK. Scale bar, 100 μm. (**B**) The protein analysis of MyHC in C2C12 cells expressing in growth or differentiation media with (+) or without (–) 1 μM p29. (**C**) Wright-Giemsa-stained C2C12 cells expressing an empty vector in growth or differentiation media with (+) or without (–) 1 μM p29 (**left**). Scale bar, 100 μm. Fusion indices were calculated in triplicate as the percentage of the total nuclei in myotubes/mm^2^ (**right**). Values are the means ± SD (*n* = 5). *P < 0.05. (**D**) Phase-contrast (**left)** and fluorescence (**right**) images of MyHC in C2C12 myoblasts expressing the empty vector (mock), myostatin, GDF11, activin A, or TGF-β1 at 7 days after differentiation with (+) or without (–) 1 μM p29. Scale bar, 100 μm. (**E**) Immunoblot analysis of MyHC protein in C2C12 cells at 7 days after differentiation with (+) or without (–) 1 μM p29 (**upper**). Densitometric analysis (**lower**). Values are the mean ± SD fold increases compared with untreated C2C12 cells expressing the empty vector (mock) (*n* = 5). *P < 0.05.

To investigate the effect of the identified inhibitory core on *in vitro* myogenesis, we synthesized a peptide, termed p29, which corresponds to the 29-amino acid inhibitory core region of mouse myostatin (^43^CAWRQNTRYSRIEAIKIQILSKLRLETAP^71^). Using a retrovirus-mediated gene transfer system [13, 14], we established C2C12 mouse myoblasts expressing various TGF-β family members including myostatin, GDF11, activin A, and TGF-β1. Mononucleated C2C12 myoblasts differentiate into multinucleated myotubes. Myotubes were stained with antibodies against myosin heavy chain (MyHC), myogenin, and creatine kinase (CK) (Fig 5A). The levels of MyHC protein were evaluated by immunoblot analysis (Fig 5B). We also quantified the fusion indices (Fig 5C). Addition of 1 μM p29 increased myotube formation and fusion Conversely, myotube formation was impaired in C2C12 myoblasts expressing TGF-β family members compared with controls expressing an empty vector (Fig 5D). Notably, p29 restored the impaired myotube formation induced by myostatin and GDF11, but not activin or TGF-β1. Consistently, p29 reversed the reduction in protein expression of MyHC in C2C12 myotubes induced by myostatin and GDF11, but not activin or TGF-β1 (Fig 5E). These findings indicate that p29 enhances myoblast differentiation by suppressing the activity of specific TGF-β family members including myostatin and its alalog GDF11 *in vitro*.

### p29 ameliorates the impaired myogenesis caused by a LGMD1C-causing mutant caveolin 3 *in vitro*

**Fig 6:**
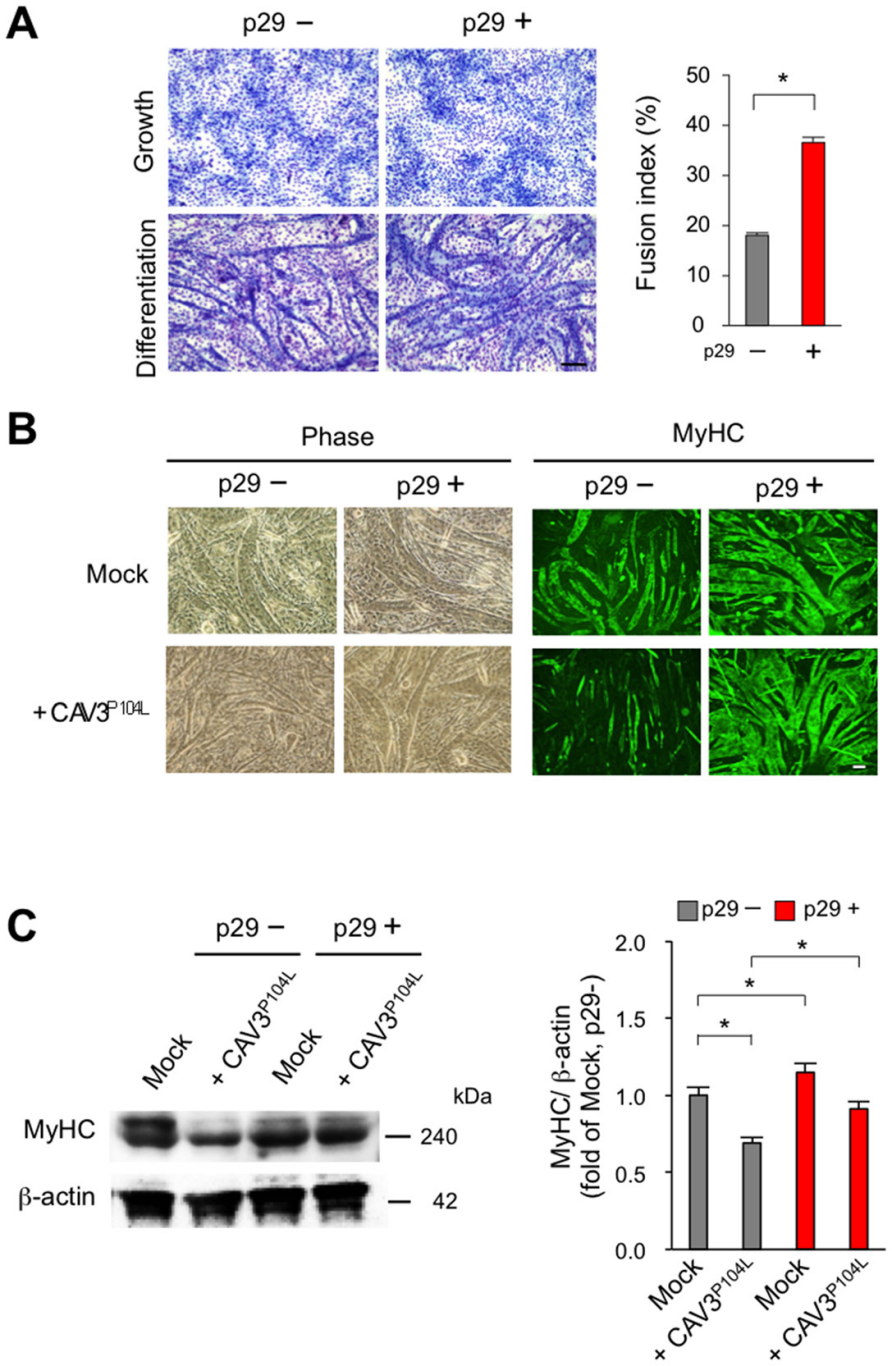
p29 restores the reduced myotube formation resulting from LGMD1C-causing mutant caveolin 3 (CAV3^P104L^). (**A**) Wright-Giemsa-stained C2C12 cells expressing LGMD1C-causing Pro104Leu mutant caveolin 3 (CAV3^P104L^) at 7 days after differentiation with (+) or without (–)1 μM p29 (**left**). Scale bar, 100 μm. Fusion indices of these cells following addition of 1 μM of p29 were calculated in triplicate as the percentage of the total nuclei in myotubes/mm^2^ (**right**). Values are the means ± SD (*n* = 5). *P < 0.05. (**B**) (**C**) Phase-contrast (**left**) and fluorescence (**right**) images of MyHC in C2C12 myoblasts expressing the empty vector (mock) or Pro104Leu mutant caveolin 3 at 7 days after differentiation with (+) or without (–) 1 μM p29. Scale bar, 100 μm. (**C**) Immunoblot analysis of MyHC and β-actin in C2C12 cells expressing the empty vector (mock) or Pro104Leu mutant caveolin 3 (CAV3^P104L^) at 7 days after differentiation with (+) or without (–) 1 μM p29 (**left**). Densitometric analysis (**right**). Values are mean ± SD fold increases compared with untreated C2C12 cells expressing the empty vector (mock) (*n* = 5). *P < 0.05.

We previously found that wild-type caveolin 3 binds to type I receptors (ALK4 and ALK5) and suppresses their activation, and that a LGMD1C-causing dominant-negative mutant caveolin 3 (CAV3^P104L^) enhances type I receptor activation by expressing these molecules using plasmid vectors in COS7 cells [10]. We retrovirally expressed the LGMD1C-causing Pro104Leu dominant-negative mutant *CAV3* cDNA in C2C12 cells to explore the molecular significance of caveolin 3 in myoblast differentiation (Fig 6). Similar to the results of expressing TGF-β family members, myoblast differentiation was impaired in C2C12 myoblasts expressing the LGMD1C-causing mutant caveolin 3 compared with empty vector-expressing controls. p29 restored the impairment in myoblast fusion (Fig 6A) and myotube formation (Fig 6B and 6C) induced by the mutant caveolin 3. Together with our previous report [10], p29 could enhance myotube formation *in vitro* by suppressing the enhanced intracellular TGF-β signals caused by the dominant-negative mutation of caveolin 3.

### p29 alleviates muscular atrophy and weakness in caveolin 3-deficient LGMD1C model mice

**Fig 7:**
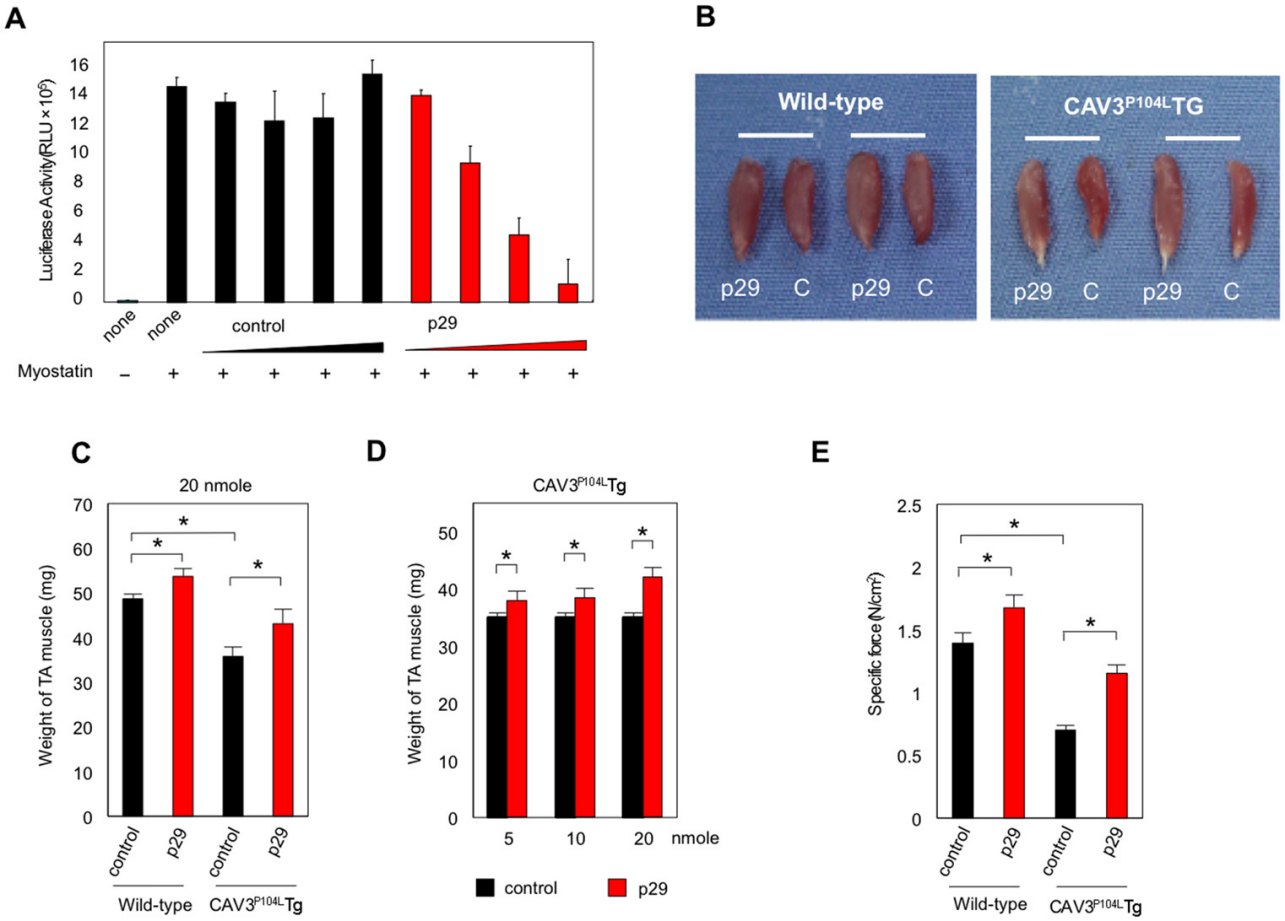
Intramuscular injection of p29 rescues muscle atrophy and weakness in caveolin 3-deficient LGMD1C model mice. (**A**) Effect of p29 on *in vitro* myostatin activity in the HEK293-(CAGA)_12_-luciferase system. Cells were stimulated with 10-ng/ml myostatin and simultaneously exposed to increasing concentrations (2, 20, 200, or 2000 nM) of p29 or albumin (control). All experiments were performed triplicate, repeatedly twice. (**B**) Appearance of TA muscles at 28 days after local injection of p29 (20 nmol) or albumin (C, control) into the ipsilateral and contralateral TA muscles of wild-type and CAV3^P104L^ Tg mice. (**C**) Weights of TA muscles injected with 20 nmol p29 or albumin in wild-type and CAV3^P104L^ Tg mice (*n* = 10). *P < 0.05. (**D**) Weights of caveolin 3-deficient TA muscles injected with different amounts of p29 or albumin (**right**, *n* = 10). *P < 0.05. (**E**) Specific force of the TA muscle in wild-type (**left**) and CAV3^P104L^ Tg (**right**) mice treated with p29 or albumin. *P < 0.05. Values are the means ± SD (*n* = 10).

Before commencement of *in vivo* experiments, we assessed the effect of p29 on *in vitro* myostatin-induced transcriptional activity in the HEK293-(CAGA)_12_-luciferase system. Consistent with our observations in co-transfection experiments, addition of p29 to the culture medium suppressed myostatin activity in a dose-dependent manner (Fig 7A). We next examined the effects of p29 in caveolin 3-deficient LGMD1C model mice (CAV3^P104L^ Tg) with elevated intramuscular TGF-β signaling that leads to muscle atrophy [10]. We injected p29 into the ipsilateral TA muscle and the same amount of albumin simultaneously into the contralateral TA muscle as a control (20 nmol, *n* = 10). After 28 days, the mice were sacrificed and their TA muscles were isolated. The TA muscles injected with p29 were larger than those injected with albumin in both wild-type littermates and caveolin 3-deficient mice, suggesting an increase in muscle mass caused by p29 (Fig 7B). On the control side, the caveolin 3-deficient mice had significantly lighter muscles compared with the wild-type mice (35.8 ± 1.8 mg *vs*. 48.6 ± 2.4 mg, *n* = 10; *P* < 0.05; Fig 7C). p29 injection significantly increased muscle weights in both caveolin 3-deficient mice and wild-type littermates (caveolin 3-deficient: 20.6% increase compared with control injection; wild-type: 10.6% increase; Fig 7C). The muscle weight increases dose-dependently by local injection of p29 into the caveolin 3-deficient muscles (*n* = 10; Fig 7D).

We also evaluated the effect of p29 on muscle performance. The specific tetanic force of untreated TA muscles was significantly reduced in CAV3^P104L^ Tg mice compared with wild-type mice (*n* = 10; Fig 7E). p29 injection increased the muscle-specific force in both CAV3^P104L^ Tg and wild-type mice. Thus, a peptide that inhibited active myostatin restored muscle-specific force in caveolin 3-deficient atrophic muscle. However, tail vein injection of the same amount of p29 once a week from 6 to 11 weeks of age showed no effect on body weight gain or muscle grip strength in caveolin 3-deficient and wild-type mice (*n* = 5; S4 Fig).

**Fig 8:**
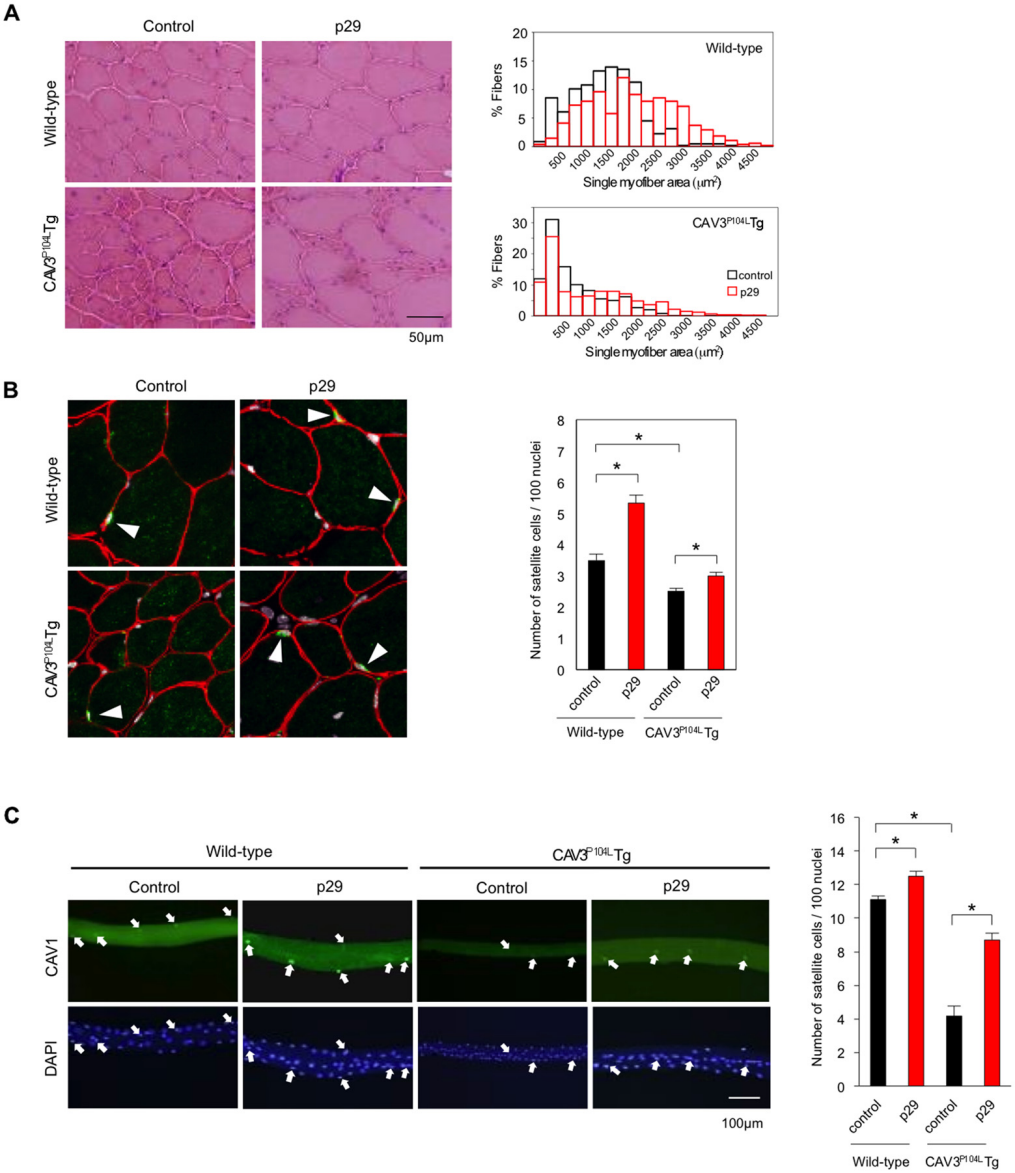
Local injection of p29 alleviates the reduction in myofiber size by restoration of the decreased numbers of satellite cells. (**A**) Histological analysis of TA muscles treated with p29 or albumin (control) in wild-type (**upper**) and CAV3^P104L^ (**lower**) mice. Scale bar, 50 μm (**left**). Distribution of SMAs in TA muscles of mice treated with p29 or albumin (**right**; *n* = 7; 250 myofibers were assessed in each mouse). (**B**) Immunohistochemical analysis of M-cadherin-positive satellite cells (green, arrows) in TA muscles of wild-type (**upper**) and CAV3^P104L^ (**lower**) mice (**left)** treated with p29 or albumin. Red indicates laminin α2 and gray indicates nuclei. Numbers of satellite cells per 100 myonuclei in TA muscles (**right**). One-thousand myonuclei were assessed in each muscle (*n* = 7). *P < 0.05. (**C**) Fluorescence images of satellite cells attached to single myofibers isolated from the TA muscles of wild-type (Wild) and CAV3^P104L^ mice treated with (+) or without (–) p29 (**left**). Mouse caveolin 1 (CAV1) was used as a marker of satellite cells (green). Nuclei were counterstained with DAPI (blue). The white arrow indicates (satellite cells). Quantification of the number of satellite cells attached to single myofibers (**right**). Numbers of satellite cells per 100 myonuclei (right). Data are expressed as the mean ± SD (*n* = 5). *P < 0.05.

Next, we examined hematoxylin and eosin-stained sections of the TA muscles from wild-type and caveolin 3-deficient mice treated with or without p29. CAV3^P104L^ Tg mouse muscles exhibited a marked reduction in the myofiber size compared with wild-type mice (*n* = 5; Fig 8A, left). p29 treatment appeared to increase the myofiber size in both wild-type and CAV3^P104L^ Tg mice. We also analyzed the single myofiber area (SMA) in each TA muscle. The SMA in TA muscles of p29-treated CAV3^P104L^ Tg mice was significantly larger than that in untreated TA muscles (1226.6 ± 497.7 μm^2^ *vs*. 752.4 ± 497.8 μm^2^, *P* < 0.05; *n* = 5; Fig 8A, right). Consistently, the SMA of TA muscles in p29-treated wild-type mice was significantly larger than that in p29-untreated wild-type mice (2102.7 ± 472.5 μm^2^ *vs*. 1504.4 ± 353.4 μm^2^, *n* = 5; *P* < 0.05; Fig 8A, right). Thus intramuscular p29 injection prevented myofiber hypotrophy in caveolin 3-deficient muscular dystrophy mice and induced postnatal myofiber hypertrophy in wild-type mice.

We further analyzed the change in fiber-type distribution by injecting p29 into TA muscles (*n* = 5; S5 Fig). We first observed that the superficial region of TA muscles consisted predominantly of fast glycolytic IIB fibers, while the deep region of TA muscles was composed of mainly IIB fibers but mixed with more abundant slow oxidative I and fast oxidative IIA fibers. In both regions, the p29 injection appeared to enlarge the size and number of IIB fibers, but not I or IIA fibers. We then measured the SMA of type IIB fibers in the deep region of TA muscles with or without p29 injection. Indeed, the SMA of type IIB fibers in p29-treated wild-type and CAV3^P104L^ Tg mouse muscles was significantly larger than that in untreated mice (3271.0 ± 823.7 μm^2^ *vs*. 2962.5 ± 476.3 μm^2^ and 2858.5 ± 428.5 μm^2^, *vs*. 1800.7 ± 224.9 μm^2^, *n* = 5; *P* < 0.05; S6 Fig). Thus, p29 treatment changed the myofiber distribution to a faster glycolytic phenotype. This fiber-type change was consistent with previously reported myostatin-null mouse muscles [22].

### Local injection of p29 increases the number of satellite cells and decreases intramuscular myostatin signaling

Satellite cells are resident stem cells between the myofiber and basal lamina in adult skeletal muscle [23]. We stained satellite cells with an anti-M-cadherin antibody in TA muscles from CAV3^P104L^ Tg and wild-type mice (*n* = 5; Fig 8B, left). In p29-untreated muscles, the number of satellite cells per 100 myonuclei was significantly reduced in caveolin 3-deficient mice compared with wild-type mice (*n* = 5; Fig 8B, right). Conversely, p29 treatment increased the numbers of satellite cells in sections of caveolin 3-deficient and wild-type mouse muscles (*n* = 5). We further stained satellite cells attached to isolated single myofibers from CAV3^P104L^ Tg and wild-type mice treated with or without p29 using an antibody against caveolin 1, another marker of satellite cells [16] (*n* = 5; Fig 8C, left). Isolated single myofiber from the CAV3^P104L^ Tg mice had a decreased number of caveolin 1-positive satellite cells compared with those from wild-type mice. Conversely, p29 injection restored the decreased number of satellite cells and myofiber size in CAV3^P104L^ Tg mice (*n* = 5; Fig 8C, right). Collectively, the synthetic peptide corresponding to the inhibitory core increased the number of resident stem cells *in vivo*.

**Fig 9:**
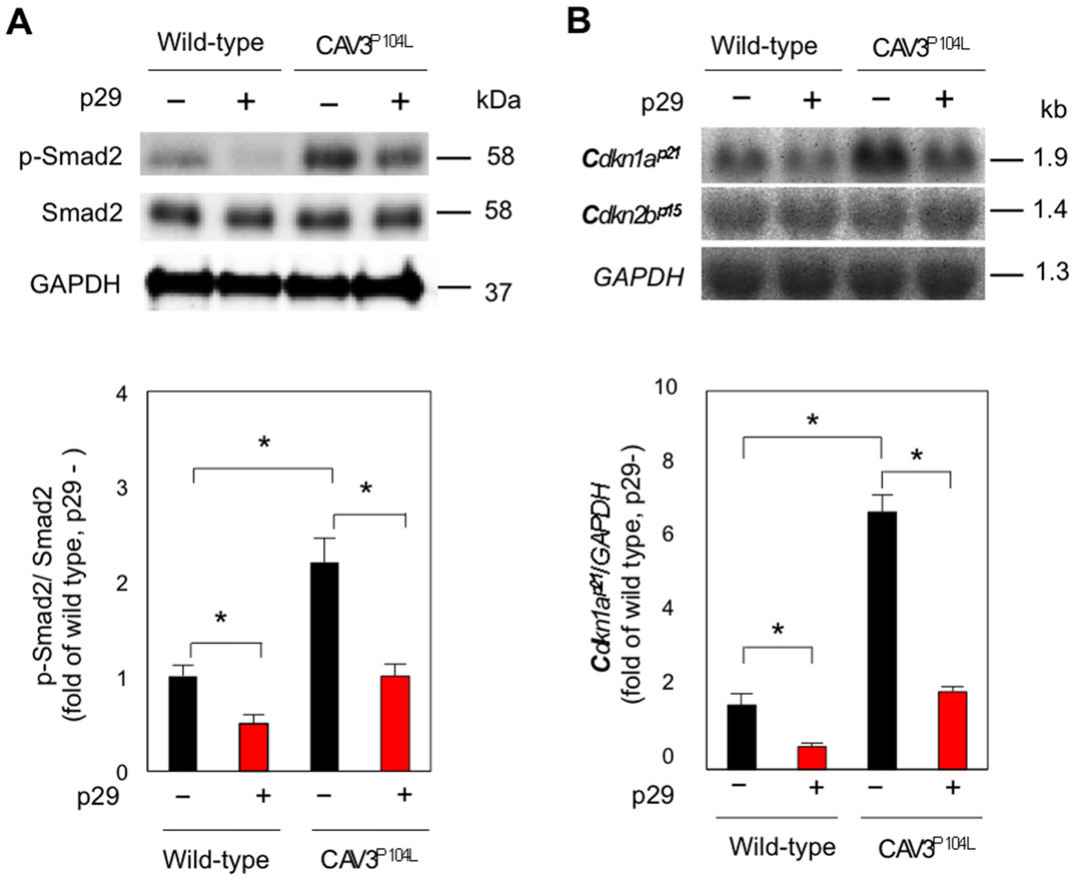
p29 inhibits activation of intramuscular myostatin signaling in caveolin 3-deficient LGMD1C model mice. Immunoblot (*n* = 5). (**A**) and northern blot (*n* = 7). (**B**) analyses of TA muscles treated with or without p29 (**upper**). Densitometric analyses (**lower**). Values are mean ± SD fold increases compared with untreated wild-type muscles. *P < 0.05.

To investigate the molecular mechanism by which p29 increases muscle mass, the levels of p-Smad2, the intracellular effector of myostatin, were analyzed in muscle homogenates (*n* = 5; Fig 9A). The amount of total Smad2 protein was comparable between wild-type and CAV3^P104L^ mice with or without p29 treatment. In contrast, the level of p-Smad2 was significantly higher in untreated muscles from CAV3^P104L^ mice. p29 treatment significantly reduced p-Smad2 levels in both wild-type and caveolin 3-deficient mice.

We previously found that cyclin-dependent kinase inhibitor p21 (*Cdkn1a* ^*p21*^) is a target gene of myostatin in mouse skeletal muscles [10,13]. To determine the effects of p29 on intramuscular myostatin signaling, the gene expression of *Cdkn1a* ^*p21*^ was examined by northern blot analysis (*n* = 7; Fig 9B). *Cdkn1a* ^*p21*^ expression was upregulated in p29-untreated caveolin 3-deficient muscles compared with untreated wild-type muscles, indicating activation of intramuscular myostatin signaling due to loss of caveolin 3. Notably, p29 injection downregulated *Cdkn1a* ^*p21*^ expression in both wild-type and CAV3^P104L^ mice. In contrast, the expression of *Cdkn2b* ^*p15*^, which we have already shown to be unaltered by myostatin [10, 13], was not affected by p29 injection. These results are consistent with our previous *in vivo* findings indicating that genetic introduction of the full-length myostatin prodomain suppresses enhanced intramuscular myostatin signaling caused by caveolin 3 deficiency [10].

## Discussion

In the current study, we revealed that the inhibitory core of the myostatin prodomain (^42^CTWRQNTKSSRIEAIKIQILSKLRLETAP^70^) resides near its N-terminus by expressing various prodomain regions as Fc fusion proteins in both HEK293 embryonic kidney cells, or A204 rhabdomyosarcoma cell. Our results are in contrast to those of a previous study in which a bacterially expressed GST-fused prodomain consisting of residues 42–98 had no inhibitory effect on *in vitro* myostatin-induced transcription [24]. The reason for this difference remains unknown, but co-expressed Fc fusion proteins might be more stable than purified GST-fusion proteins in the specific type of assay. Alternatively, peptides expressed in mammalian cells, but not prokaryotic cells, might achieve the properly folded state.

The identified inhibitory core of the myostatin prodomain includes an AH that is evolutionarily conserved among other TGF-β family members. Of note, a previous mutational analysis demonstrated that the AH in the TGF-β1 prodomain is required for both its ligand-binding and inhibitory capacities [18]. Recent three-dimensional crystallography analyses of TGF-β1 predicted that the neighboring portions of the AH (the N-terminal RC, and C-terminal LL) are located extremely close to their type I and type II receptors, and appear to shield the ligand from binding to its specific receptors [19, 20]. We demonstrated for the first time that the inhibitory core of the myostatin prodomain can interact with the type I and II receptors as well as the ligand by co-localization and co-immunoprecipitation experiments. We next found that combined deletion of portions of the inhibitory core (the RC, AH, or LL) in the full-length prodomain removes all inhibitory effects on myostatin activation. Moreover, we found that the myostatin inhibitory core specifically suppressed myostatin and its analog, GDF11, which shares identical type I and II receptors [25,26]. However, the myostatin inhibitory core did not suppress TGF-β1 or activin A, both of which have a similar AH structure but bind to different receptors [2, 5]. Additionally, the inhibitory core peptide (p29) enhanced restoration of the impaired myoblast differentiation induced by myostatin and GDF11, but not activin or TGF-β1. Consistent with our results, Thies et al. reported that the full-length myostatin prodomain suppresses *in vitro* transcriptional activities induced by myostatin and GDF11, but not activin A, in A204 cells [17]. Moreover, ligand-receptor binding of radiolabeled myostatin on the cell surface is dose-dependently suppressed by the full-length myostatin prodomain in L6 rat myoblast cells [17]. Taken together, our data suggest a novel concept that the identified inhibitory core of the myostatin prodomain has both binding and inhibitory effects on its ligand by coordinate interactions with type I and II membrane receptors. As observed in other crystal structure studies [27–29], the corresponding inhibitory core in the prodomain of certain TGF-βs are likely to co-locate approximately with its receptors, thus functioning to prevent an individual ligand from binding their two specific receptors on the cell surface.

Intramuscular injection of p29 increased muscle mass and strength by increasing the number of muscle precursor satellite cells in wild-type mice. Consistent with the *in vitro* results indicating that p29 reversed the impaired myogenic differentiation resulting from the LGMD-causing mutant caveolin 3, local injection of p29 alleviated muscle atrophy in TGF-β-activated muscles in LGMD1C model mice by restoration of enhanced TGF-β signaling *in vivo*. Thus, we have provided the first proof-of-concept of a synthetic peptide drug that blocks myostatin signaling to reverse muscle atrophy *in vivo*.

Several inhibitors that antagonize the activation of myostatin, a crucial negative regulator of muscle mass, have been developed recently to treat muscle-wasting disorders. These inhibitors include neutralizing antibodies against the myostatin ligand [30], its type II receptors [31], and type II decoy receptors [10,32], and a small molecule that inhibits type I receptors [13]. Compared with these nonphysiological blockers that target signal transduction molecules, the inhibitory core peptide for the myostatin prodomain may be advantageous not only because it is a circulating physiological blocker of myostatin ligand in the inactive complex, but also because it disturbs ligand-receptor binding specifically on the cell surface. In the present study, intravenous injection of p29 failed to ameliorate muscle atrophy in TGF-β-activated LGMD1C model mice. The dosage of p29 for systemic administration could be insufficient to increase muscle mass. Alternatively, p29 peptide could be unstable against proteolysis when intravenously administered. Indeed, the bacterially expressed GST-fused prodomain peptide consisting of the N-terminal half is reported to be more unstable than that consisting of the C-terminal half [24]. We have recently revealed the precise peptide structure of the inhibitory core and its high affinity for the myostatin ligand [33]. Based on these structural data, further modifications of the degradation-competent amino acid architecture and the development of effective delivery methods to target skeletal muscle will advance rational and potent therapies incorporating synthetic myostatin-blocking peptides.

## Supporting Information

S1 Fig Immunoblot analysis of whole cell extracts using an anti-human Fc antibody (Upper) and the corresponding Ponceau S staining (lower). Left lane is a protein size standard.
(TIF)

S2 Fig Luciferase activities in A204 rhabdomyosarcoma cells. (A) Truncation and deletion constructs of human myostatin prodomain:human Fc fusion proteins (left). Percentage inhibitory effect of each construct on myostatin activity in comparison with the full-length prodomain (f-Pro, right). (B) Recombinant myostatin-induced transcriptional activity in A204 cells co-transfected with a pGL3-(CAGA)_12_-luciferase reporter gene, pCMV-β-Gal, and various prodomain region:Fc fusion constructs. Values are the mean ± SD (*n* = 6). RLU, relative luminescence units.
(TIF)

S3 Fig Co-localization (A) and co-immunoprecipitation (B) of the inhibitory core (IC) of the myostatin prodomain with its analog, GDF11. (A) COS-7 cells expressing the FLAG-tagged IC of myostatin and HA-tagged GDF11 (prodomain+ligand, or ligand). Scale bar, 20 μm. (B) Whole cell extracts (WCE) were immunoprecipitated with anti-FLAG or anti-HA agarose, then immunoblotted using anti-FLAG or anti-HA antibodies, respectively.
(TIF)

S4 Fig Growth curve and grip strength following systemic p29 injection. p29 (20 nmol) was injected intravenously once a week from 6 to 11 weeks of age into wild-type and caveolin 3-deficient mice (*n* = 5). Data are expressed as the mean ± SD (*n* = 5).
(TIF)

S5 Fig Fiber type distribution of TA muscles treated with (+) or without (–) p29. Hematoxylin and eosin-, NADH-TR-, fast glycolytic type IIB MyHC-, fast oxidative type IIA MyHC-, and slow oxidative type MyH-stained sections showed the superficial and deep regions of TA muscles in wild-type (upper) and CAV3^P104L^ Tg (lower) mice treated with (+) or without (–) p29. Fast (extensor digitorum longus, EDL) and slow (soleus) muscle sections from wild-type mice were used as a staining control. Scale bar, 50 μm.
(TIF)

S6 Fig Histogram of the SMA of fast glycolytic type IIB fibers in the deep region of the TA muscles. The SMA in type IIB fast glycolytic fibers of wild-type (left) and CAV3^P1`4L^ Tg (right) mice treated with (red) or without (white) p29 (*n* = 5; 125 myofibers were assessed in each mouse).
(TIF)
