# Supplementary figures and images for: The Inhibitory Core of the Myostatin Prodomain: Its Interaction with Both Type I and II Membrane Receptors, and Potential to Treat Muscle Atrophy
Source: PLoS One. 2015 Jul 30;10(7):e0133713. doi: 10.1371/journal.pone.0133713 (PMC4520684; doi:10.1371/journal.pone.0133713)

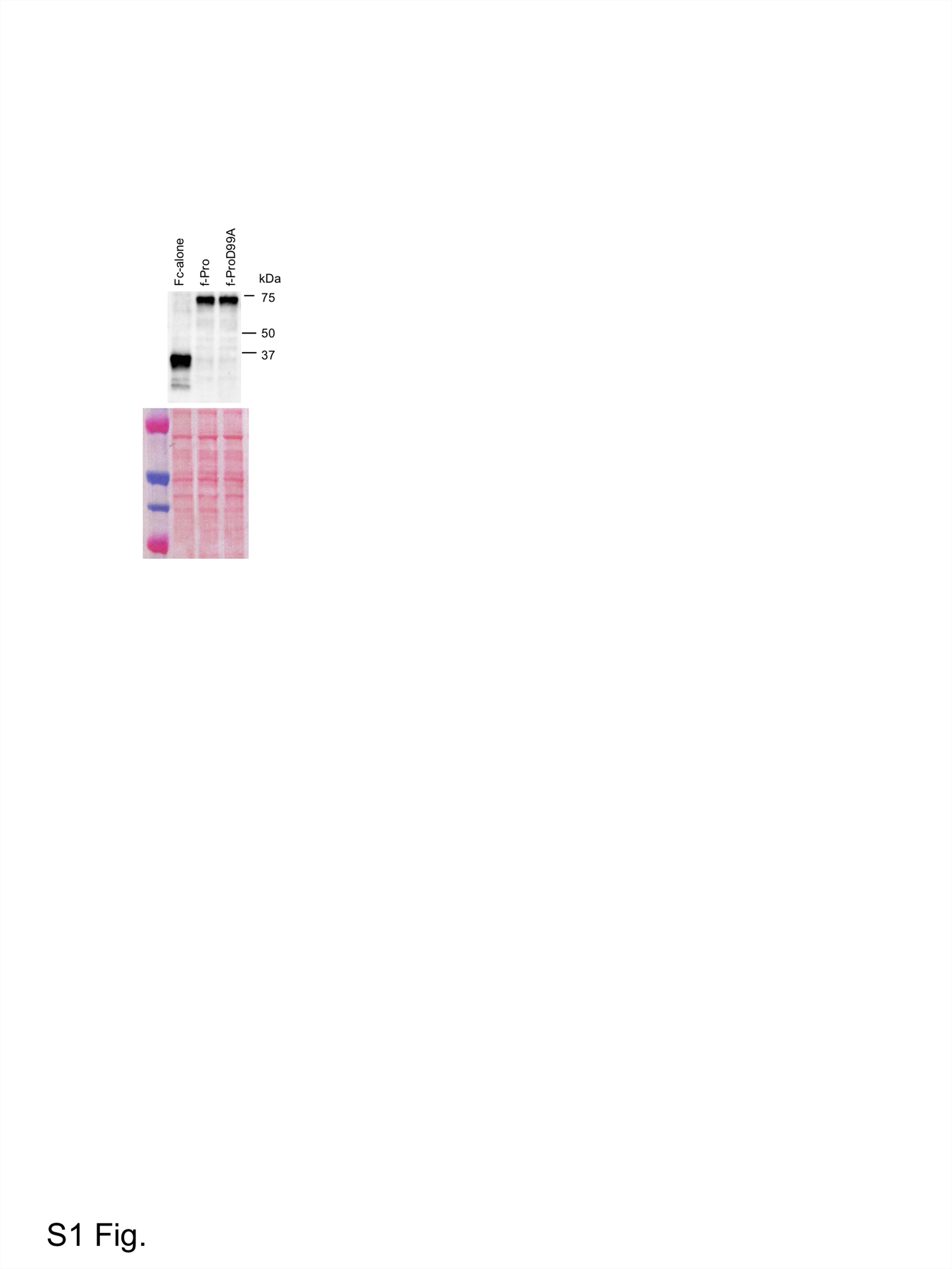

Supplement: S1 Fig — Left lane is a protein size standard. (TIF) [file pone.0133713.s001.tif]

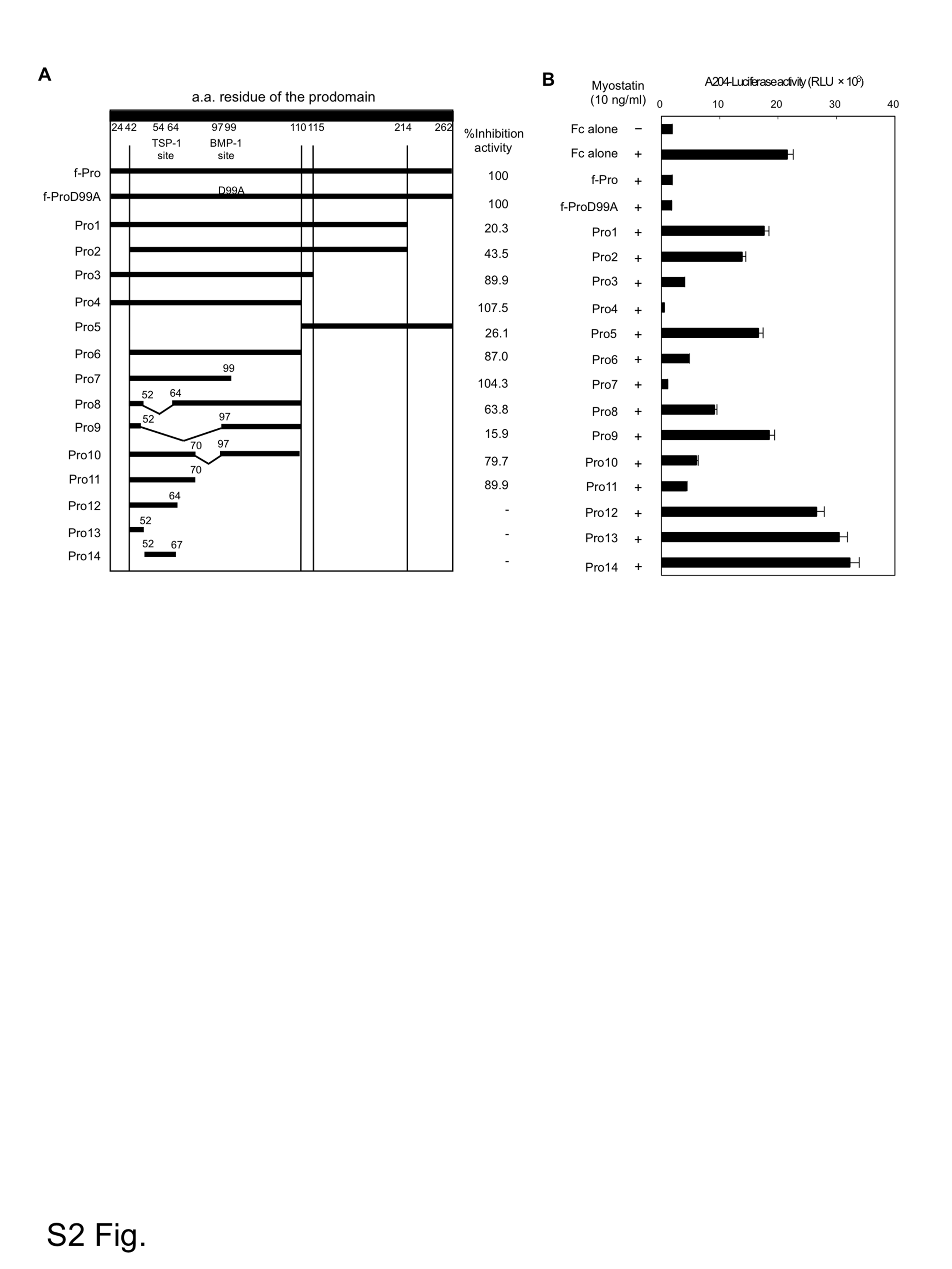

Supplement: S2 Fig — (A) Truncation and deletion constructs of human myostatin prodomain:human Fc fusion proteins (left). Percentage inhibitory effect of each construct on myostatin activity in comparison with the full-length prodomain (f-Pro, right). (B) Recombinant myostatin-induced transcriptional activity in A204 cells co-transfected with a pGL3-(CAGA)12-luciferase reporter gene, pCMV-β-Gal, and various prodomain region:Fc fusion constructs. Values are the mean ± SD (n = 6). RLU, relative luminescence units. (TIF) [file pone.0133713.s002.tif]

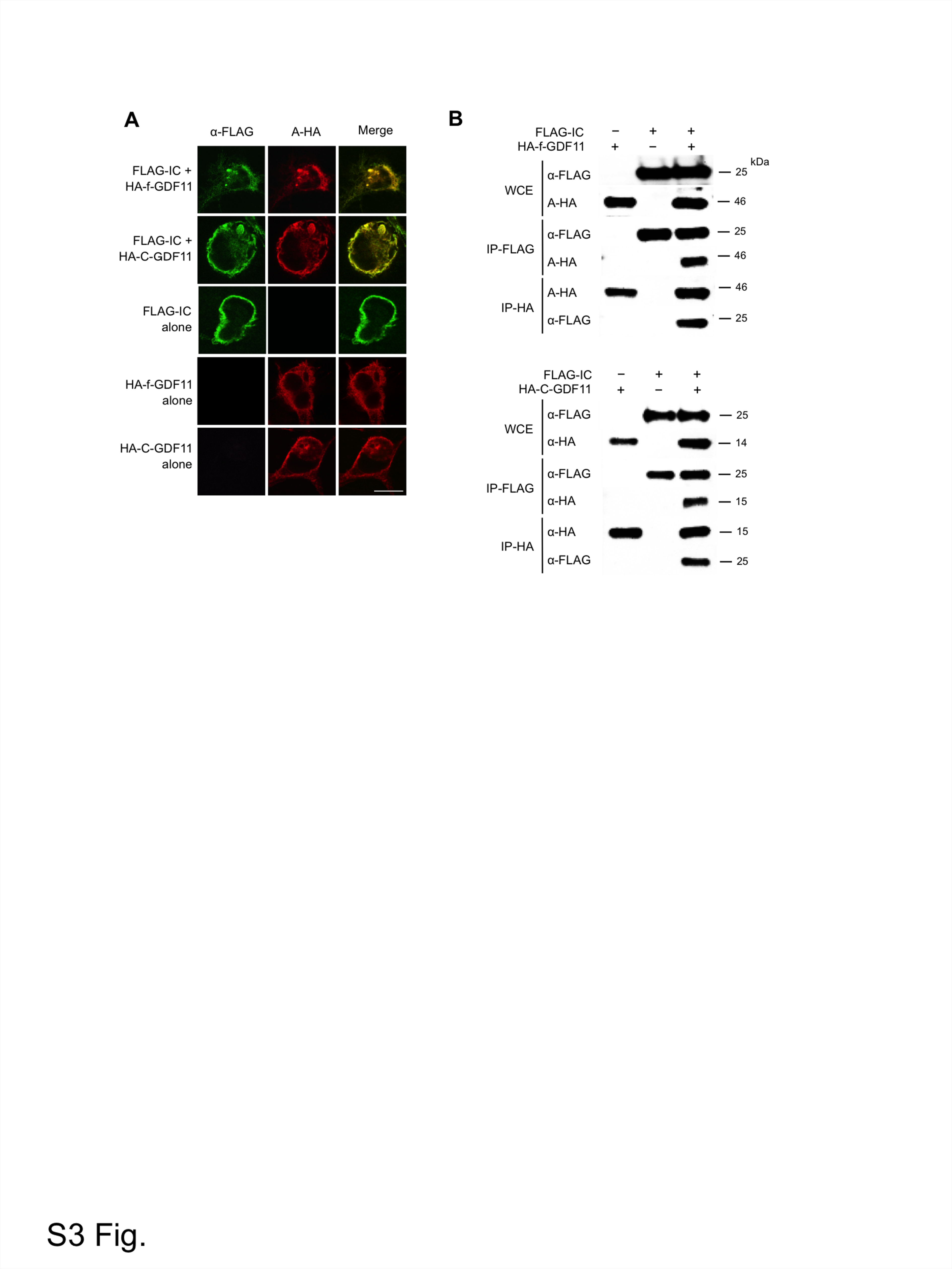

Supplement: S3 Fig — (A) COS-7 cells expressing the FLAG-tagged IC of myostatin and HA-tagged GDF11 (prodomain+ligand, or ligand). Scale bar, 20 μm. (B) Whole cell extracts (WCE) were immunoprecipitated with anti-FLAG or anti-HA agarose, then immunoblotted using anti-FLAG or anti-HA antibodies, respectively. (TIF) [file pone.0133713.s003.tif]

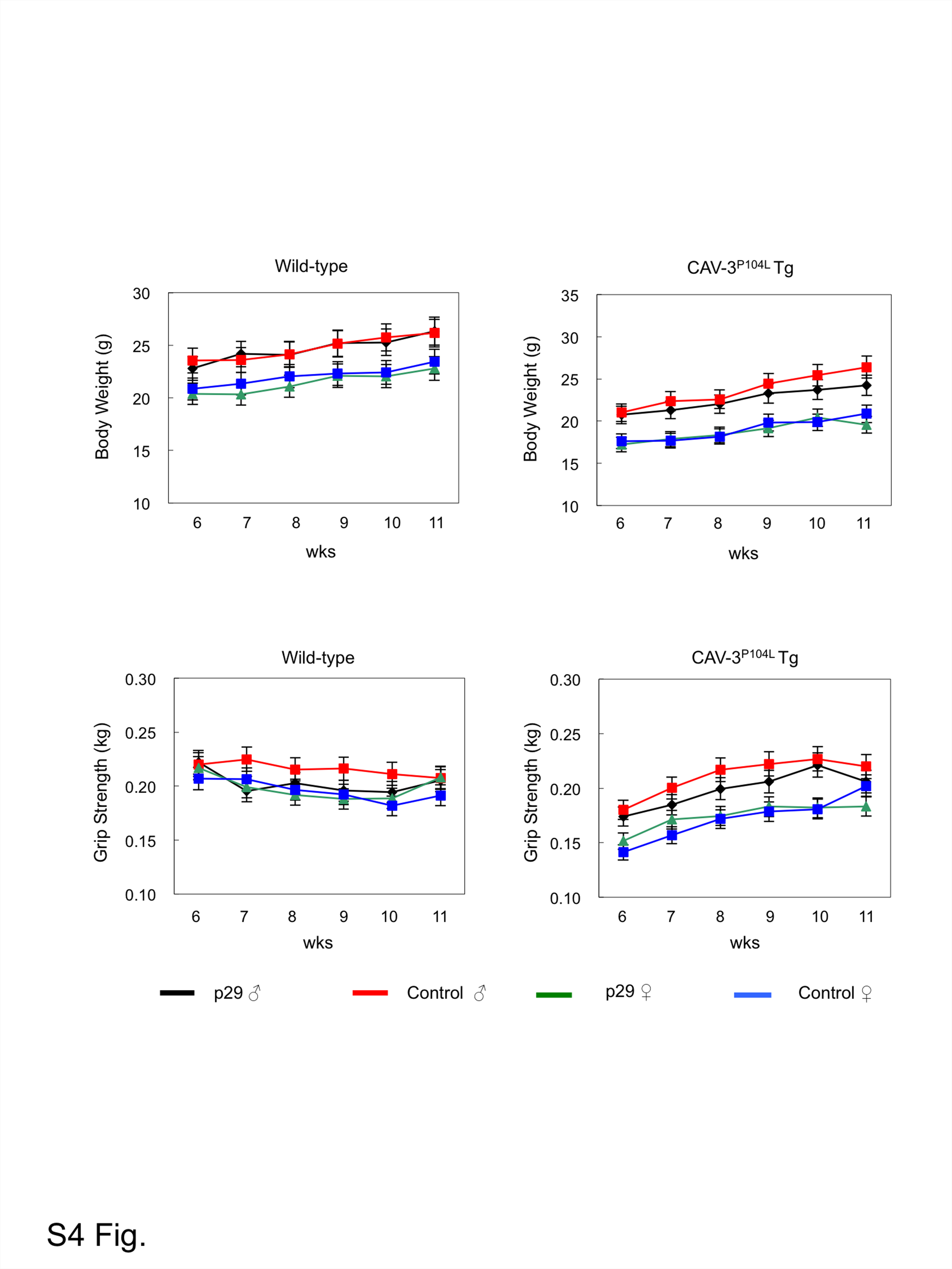

Supplement: S4 Fig — p29 (20 nmol) was injected intravenously once a week from 6 to 11 weeks of age into wild-type and caveolin 3-deficient mice (n = 5). Data are expressed as the mean ± SD (n = 5). (TIF) [file pone.0133713.s004.tif]

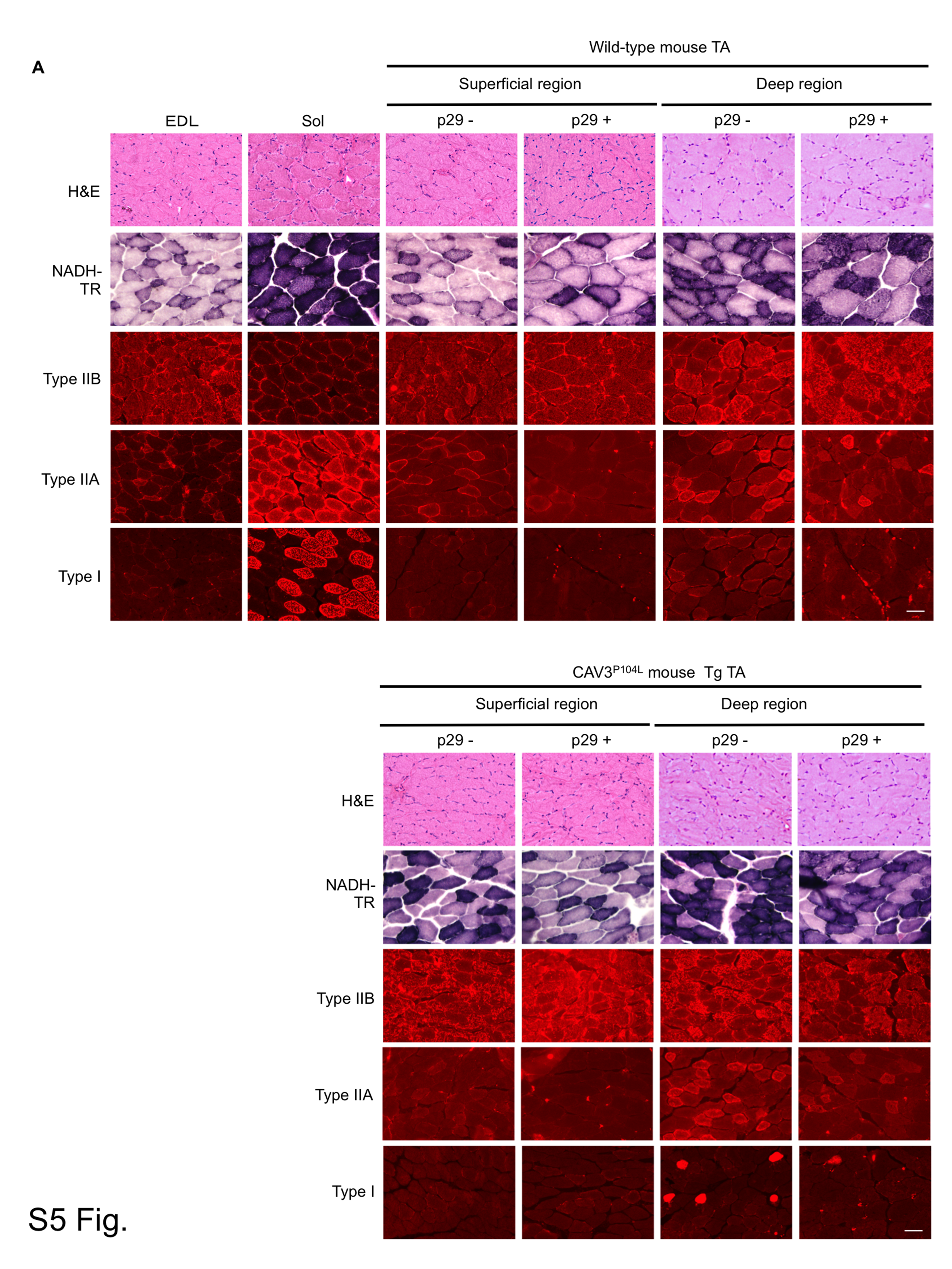

Supplement: S5 Fig — Hematoxylin and eosin-, NADH-TR-, fast glycolytic type IIB MyHC-, fast oxidative type IIA MyHC-, and slow oxidative type MyH-stained sections showed the superficial and deep regions of TA muscles in wild-type (upper) and CAV3P104L Tg (lower) mice treated with (+) or without (–) p29. Fast (extensor digitorum longus, EDL) and slow (soleus) muscle sections from wild-type mice were used as a staining control. Scale bar, 50 μm. (TIF) [file pone.0133713.s005.tif]

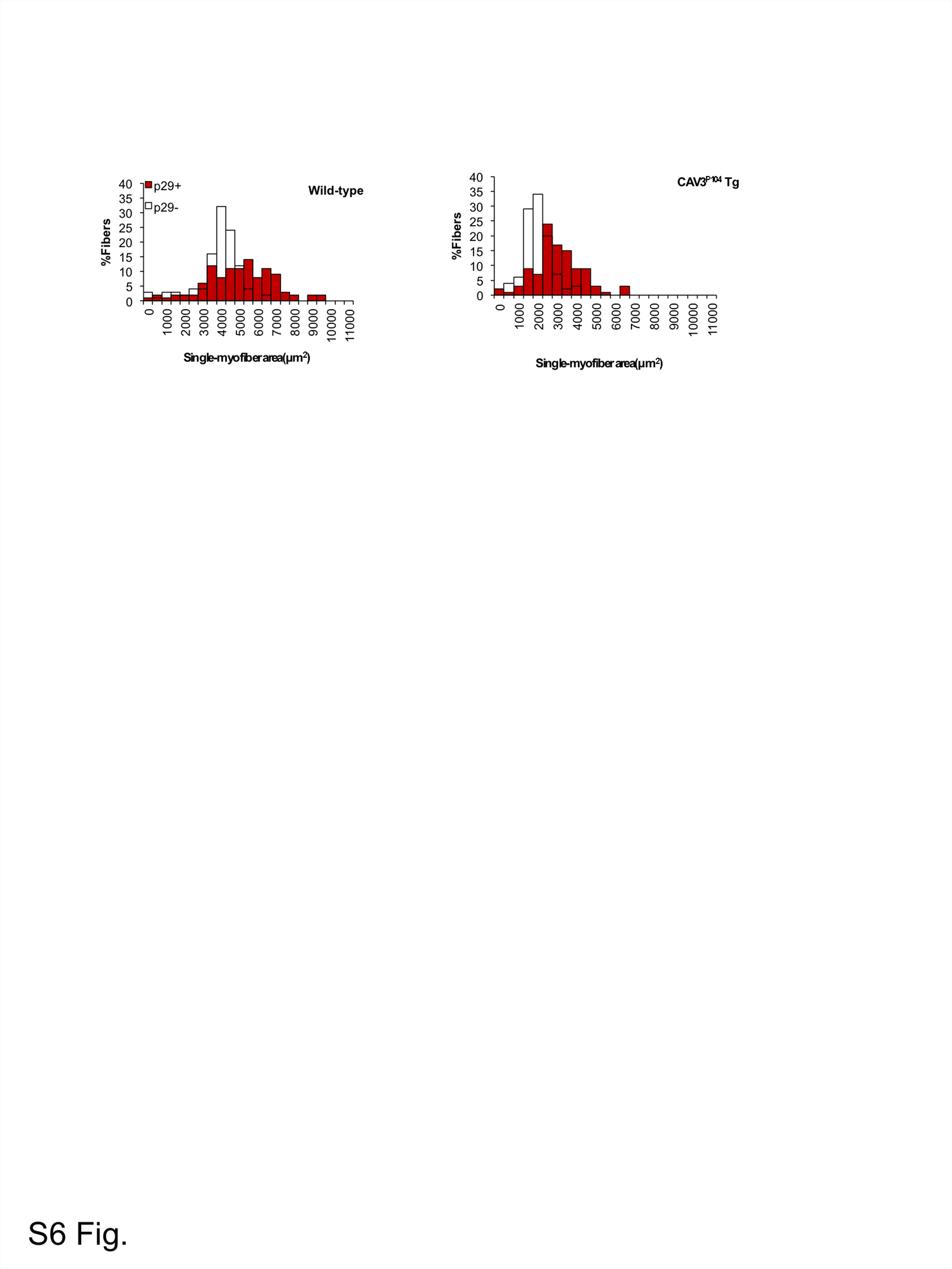

Supplement: S6 Fig — The SMA in type IIB fast glycolytic fibers of wild-type (left) and CAV3P1`4L Tg (right) mice treated with (red) or without (white) p29 (n = 5; 125 myofibers were assessed in each mouse). (TIF) [file pone.0133713.s006.tif]
